# Evolutionary conserved role of eukaryotic translation factor eIF5A in the regulation of actin-nucleating formins

**DOI:** 10.1038/s41598-017-10057-y

**Published:** 2017-08-29

**Authors:** Verónica Muñoz-Soriano, Ana Domingo-Muelas, Tianlu Li, Esther Gamero, Alexandra Bizy, Isabel Fariñas, Paula Alepuz, Nuria Paricio

**Affiliations:** 1 Departamento de Genética, Universidad de Valencia, 46100 Burjassot, Spain; 2 Departamento de Biología Celular & Centro de Investigación Biomédica en Red sobre Enfermedades Neurodegenerativas (CIBERNED), Universidad de Valencia, 46100 Burjassot, Spain; 3 Departamento de Bioquímica y Biología Molecular, Universidad de Valencia, 46100 Burjassot, Spain; 4 Estructura de Recerca Interdisciplinar en Biotecnologia i Biomedicina (ERI BIOTECMED), Universidad de Valencia, 46100 Burjassot, Spain; 5 Present Address: Institut d’Investigació Biomèdica de Bellvitge (IDIBELL), 08908 Hospitalet de Llobregat, Spain

## Abstract

Elongation factor eIF5A is required for the translation of consecutive prolines, and was shown in yeast to translate polyproline-containing Bni1, an actin-nucleating formin required for polarized growth during mating. Here we show that *Drosophila* eIF5A can functionally replace yeast eIF5A and is required for actin-rich cable assembly during embryonic dorsal closure (DC). Furthermore, Diaphanous, the formin involved in actin dynamics during DC, is regulated by and mediates eIF5A effects. Finally, eIF5A controls cell migration and regulates Diaphanous levels also in mammalian cells. Our results uncover an evolutionary conserved role of eIF5A regulating cytoskeleton-dependent processes through translation of formins in eukaryotes.

## Introduction

Cytoskeletal remodeling in response to signal transduction pathways is critical to many cellular processes. Actin-nucleating proteins of the formin and Arp2/3 protein families mediate the first step of filament assembly, contributing to cell shape, polarity, adhesion, and migration among others. Formins in particular allow the initiation and growth of linear filaments through their conserved actin polymerization domains FH1 and FH2^1^. Interestingly, FH1 domains contain long stretches of consecutive prolines, which decrease translation efficiency due to prolines being poor acceptors and donors during peptide bond formation thus causing ribosome stalling^2^. Therefore, the FH1 domain may have an impact in how formin protein levels are regulated and hence on how the cell responds to cytoskeletal demands.

The eukaryotic translation initiation factor 5A (eIF5A), and its homolog in prokaryotes EF-P, has recently been described to alleviate ribosome stalling during translation of proline stretches (polyPro motifs)^3–5^. Remarkably, eIF5A is the only known protein to contain the polyamine-derived amino acid hypusine, a post-translational modification that is essential for its role in translation (reviewed in ref. 6). Current data suggest that eIF5A could bind stalled ribosomes with a free E-site, and then hypusinated eIF5A would facilitate the formation of a new peptide bond^7^. Recent genomic analyses of ribosome dynamics have demonstrated that ribosomes stall at polyPro motifs in the absence of eIF5A. These studies also show that eIF5A promotes peptide bond formation at many other specific tripeptide motifs and facilitates translation termination globally^8, 9^. eIF5A is essential in all eukaryotes and has been implicated in cell proliferation and cytoskeleton remodeling processes^10^. For example, in yeast and trypanosome the absence of activated eIF5A causes changes in actin dynamics and cell shape^11–13^. Moreover, overexpression of eIF5A isoforms has been observed in several tumors (e.g. pancreatic, hepatic, colon, lung and ovarian) and promotes cell migration, invasion and cancer metastasis^10, 14^.

Recently, we showed that the depletion of the essential *Saccharomyces cerevisiae* (Sc) *TIF51A* gene, one of the two genes encoding Sc eIF5A, causes a sterile phenotype due to reduced synthesis of the Bni1 formin. Translation of the polyPro motifs located in the FH1 domain of Bni1 required eIF5A; consequently, eIF5A conditional mutants in yeast haploid cells were unable to grow a mating projection, termed shmoo, towards a pheromone gradient during mating^15^. Despite the functional connection between eIF5A and translation of polyPro-containing proteins in yeast, it is unclear whether eIF5A can regulate cytoskeletal remodeling through the translation of formins in other systems.

In this study, we identified a role of *Drosophila* eIF5A (Dm eIF5A) in dorsal closure (DC), the last major morphogenetic rearrangement during embryogenesis. DC involves the formation of an actomyosin contractile supracellular cable together with the migration, spreading and fusion of lateral epidermal cellular sheets^16, 17^. We have found that Dm eIF5A is the functional homolog of Sc eIF5A and its depletion in *Drosophila* causes defects in actomyosin cable formation in the embryonic epidermis during DC. Our results also revealed that eIF5A regulates the protein levels of Diaphanous (Dia), and that this formin is required during DC downstream of eIF5A. Lastly, depletion of active eIF5A decreases the levels of Dia and migration in mouse neural stem cells (NSCs). Together, our results indicate an evolutionary conserved function of eIF5A in the translation of actin-nucleating formins with important consequences on the regulation of cytoskeleton-mediated processes from yeast to mammals.

## Results and Discussion

### The translational regulator eIF5A is required for actin cable assembly during *Drosophila* DC

**Figure 1:**
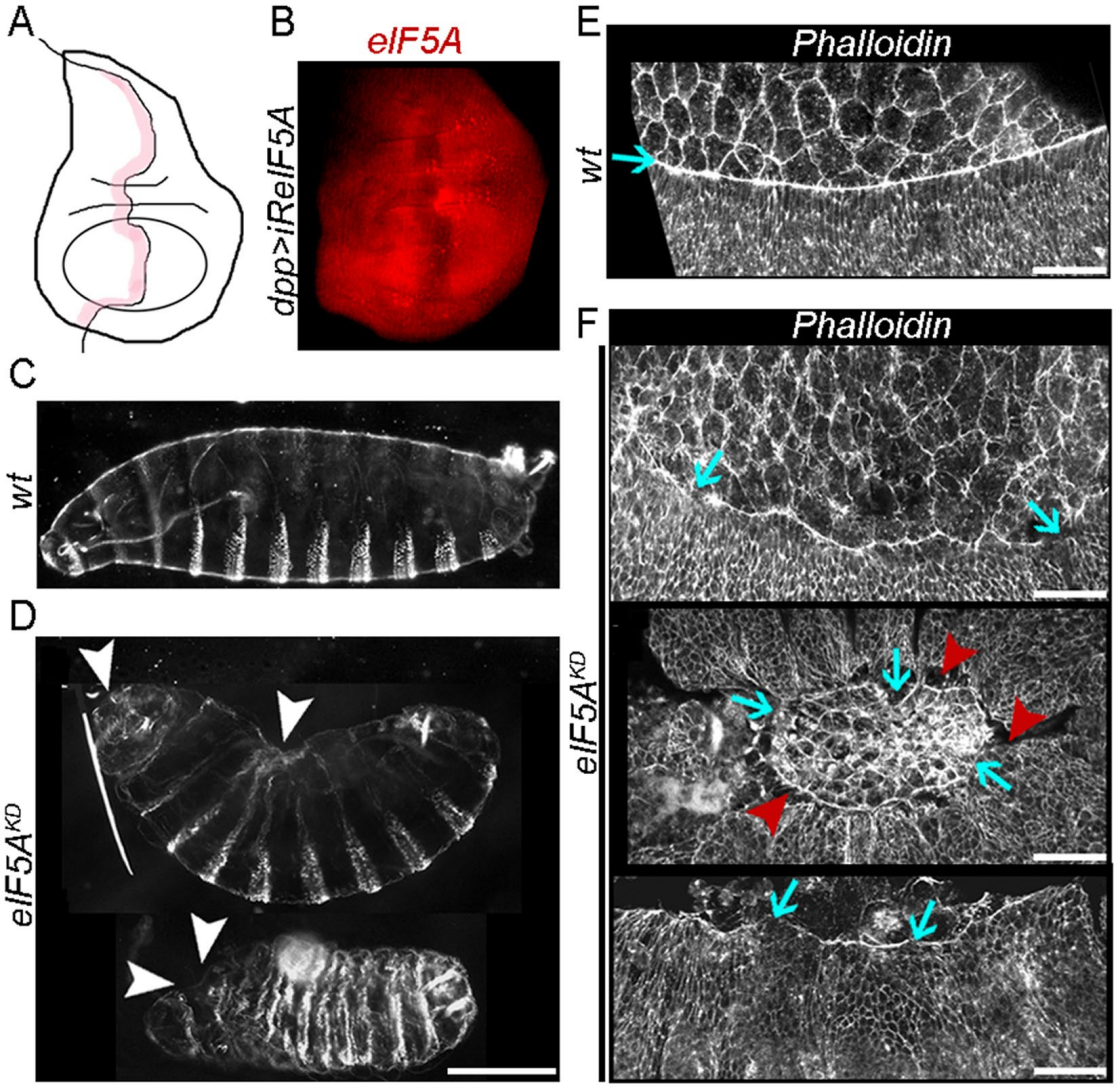
Knockdown of Dm *eIF5A* in the embryonic epidermis produces defects in DC and actin cable assembly. (**A**) Schematic representation of the anterior-posterior (AP) compartment boundary of a *Drosophila* wing imaginal disc (shaded in pink). (**B**) Anti-eIF5A antibody staining of a *dpp*-GAL4/UAS-*iReIF5A* wing imaginal disc, in which *eIF5A* knockdown is induced in the AP boundary. (**C,D**) Dark-field micrographs of representative (**C**) wild-type and (**D**) *eIF5A* mutant embryos (*eIF5A* ^*KD*^). Anterior is to the left, dorsal is up. White arrowheads point to holes or not properly sealed areas in the anterior and dorsal parts of the embryo. (**E,F**) Confocal images of the lateral epidermis, DME cells and AS cells of representative stage 13–14 (**E**) control and (**F**) *eIF5A* ^*KD*^ embryos stained with phalloidin. Blue arrows point to the actomyosin cable in DME cells, which is irregular and discontinuous in *eIF5A* mutants. Detachment of DME cells from AS cells is frequently observed in mutant embryos (red arrowheads in **F**). Scale bars: 100 µm in **C,D**; 25 µm in **E,F**.

During *Drosophila* embryogenesis, the dorsal epidermis appears discontinuous due to germ band retraction and is transiently covered by an extraembryonic tissue, the amnioserosa (AS). The hole is subsequently sealed by a complex process called DC, which involves the formation of an actomyosin contractile cable at the leading edges of the dorsal-most epidermal (DME) cells, the emission of cellular protrusions from opposite sides of the epidermis that interdigitate and zip the dorsal opening at its corners, and the apical constriction and apoptosis of AS cells that facilitates the occlusion of the hole. As a result, the two lateral epidermal sheets converge towards the dorsal midline while the AS reduces its surface area until it disappears inside the embryo^16, 17^. The *cabut* (*cbt*) gene encodes a transcription factor with high similarity to the vertebrate TIEG (TGF-β-inducible early-response genes) proteins and is involved in several key events during DC including actin dynamics^18, 19^. However, its direct target genes during this process are unknown. Using genome-wide ChIP-on-chip analysis for Cbt binding sites in extracts from 9–14 h embryos we identified *eIF5A* as a Cbt putative target gene (V.M.-S. and N.P., in preparation). Reduced *eIF5A* function affects cell growth and causes autophagy in several *Drosophila* tissues and increases polysome size in S2 cells^20^, suggesting a potential role in translation elongation. However, the role of the only Dm *eIF5A* gene in DC has never been investigated. To clarify this issue, we reduced the expression of Dm *eIF5A* specifically in the embryonic epidermis using a UAS-*iReIF5A* RNAi line and the *arm*-GAL4 or *69B*-GAL4 epidermal drivers (Fig. 1A,B) which resulted in late embryonic lethality. Cuticle preparations revealed that most *eIF5A* mutant (*eIF5A* ^*KD*^) embryos displayed DC defects with holes in the dorsal and anterior epidermal regions (Fig. 1C,D). Phalloidin stainings were performed to examine the integrity of the actin cable at the leading edge of DME cells and revealed an irregular and discontinuous distribution of actin in *eIF5A* mutants (Fig. 1E,F). Defective attachments between AS and epidermal cells, as well as misalignments at the dorsal midline, were also observed (Fig. 1F). Taken together, these results indicated that Dm *eIF5A* plays an essential role in actomyosin organization during embryogenesis.

### *Drosophila* eIF5A is a functional homolog of yeast eIF5A by rescuing the translation of polyPro-containing formins in yeast *TIF51A* mutants

**Figure 2:**
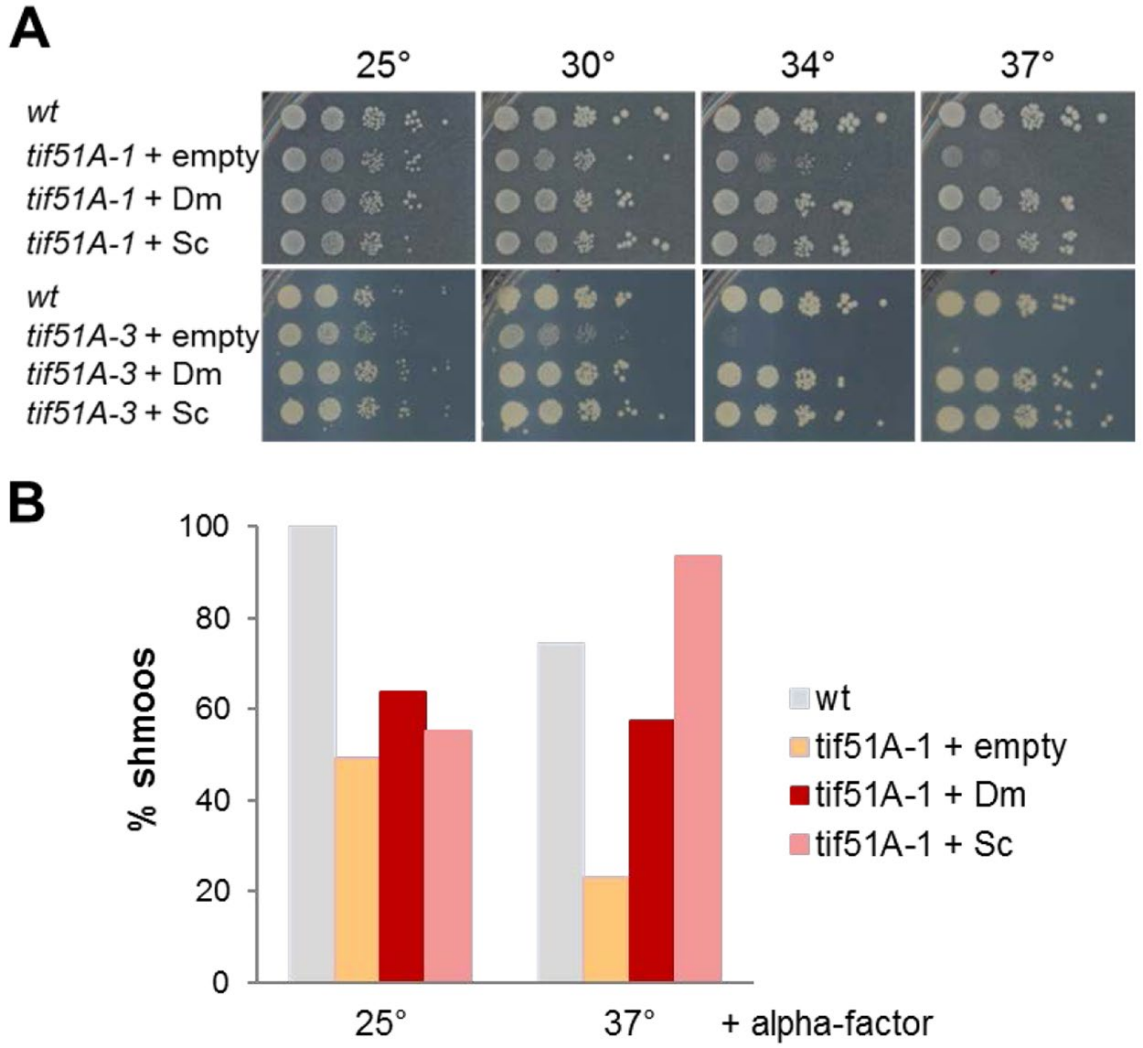
Heterologous expression of Dm eIF5A restores growth and shmoo formation of Sc eIF5A mutant cells. (**A**) Wild-type and *tif51A-1* or *tif51A-3 S. cerevisiae* mutants containing empty plasmid or plasmids expressing Dm eIF5A or Sc eIF5A genes were grown at permissive temperature (25 °C) until exponential phase and then plated (10-fold serial dilutions) onto YPD medium and incubated at the indicated temperatures. (**B**) Same yeast strains and transformants described in (**A**) were grown until exponential phase and then treated or not with 10 μg/ml α-factor for 2 h. Yeast cells were then maintained at 25 °C or transferred to 37 °C for 4 h and DIC images were reported. Quantifications of percentage of cells containing shmoos in samples treated with α-factor are indicated. Approximately 200 cells were manually counted for each sample from at least two independent experiments.

Human eIF5A can restore growth in yeast cells lacking their own eIF5A^21^. To investigate whether Dm eIF5A was also a functional homolog of Sc eIF5A, we cloned the Dm *eIF5A* cDNA and the ORF of the *TIF51A* yeast gene in yeast plasmids under the control of the constitutive TEF promoter and with the *CYC1* terminator. We then transformed these plasmids, or an empty plasmid as control, in two temperature sensitive yeast mutants, *tif51A-1* and *tif51A-3*, which contain one (P83S) or two (C39Y, G118D) amino acid substitutions, respectively, and are unable to grow at restrictive temperature (37 °C)^22, 23^. Yeast mutants transformed with plasmids containing either Dm eIF5A or Sc eIF5A exhibited restored growth at the restrictive temperature (Fig. 2A). With regards to the defect in pheromone-induced shmoo formation observed in eIF5A-depleted yeast haploid cells, *tif51A-1* mutants containing empty, Dm eIF5A or Sc eIF5A plasmids showed reduced shmoo formation under permissive temperature, suggesting that the Tif51A-1 mutated version of yeast eIF5A plays a dominant role. However, upon the introduction of Sc eIF5A and Dm eIF5A shmoo formation was restored to full or almost full (77% for Dm eIF5A) wild-type levels at 37 °C (Fig. 2B). These data indicated that Dm eIF5A is a functional homolog of yeast eIF5A, allowing normal growth and pheromone-induced polarized growth in eIF5A-deficient yeast cells.

**Figure 3:**
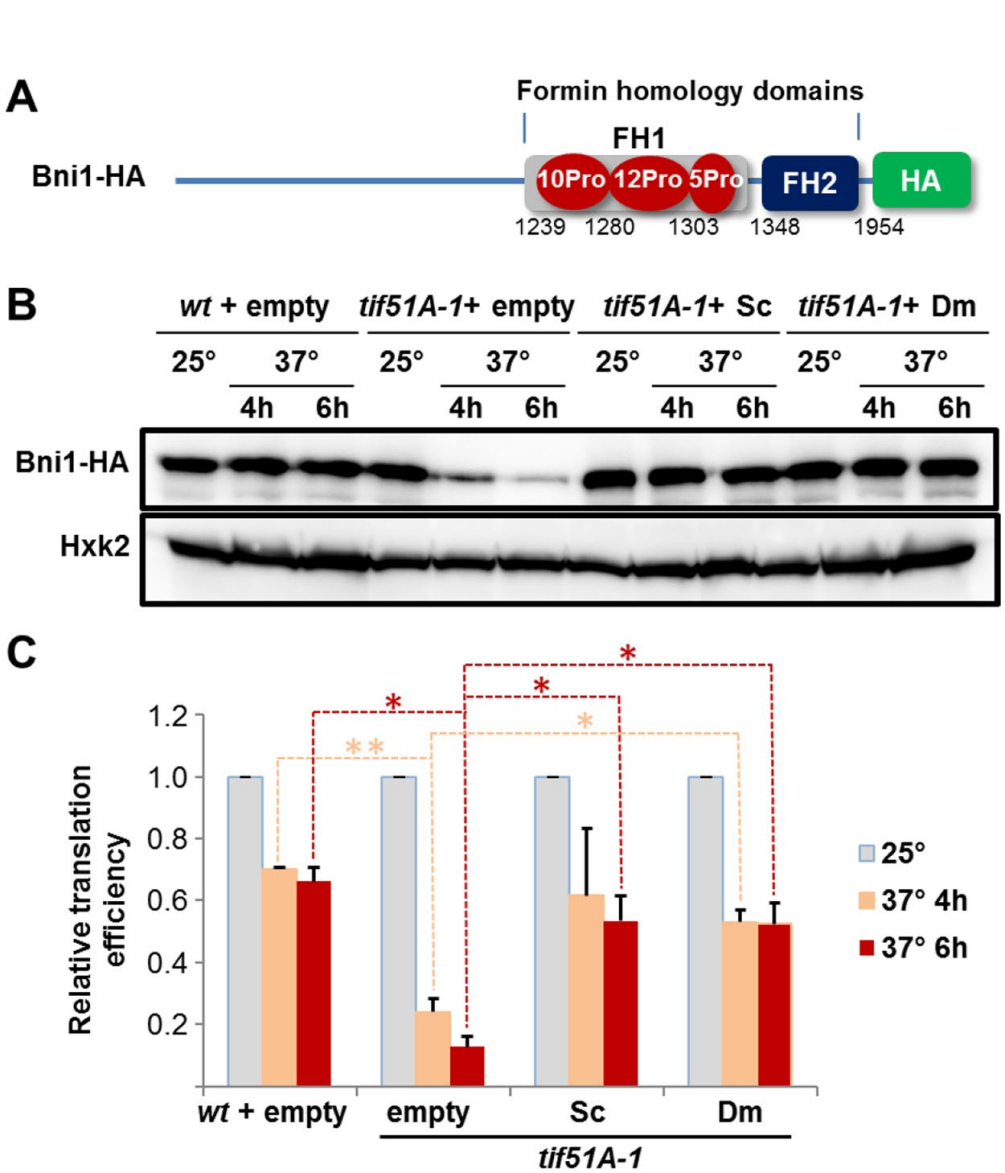
Heterologous expression of Dm eIF5A allows translation of the yeast polyPro formin *BNI1* in Sc eIF5A-depleted cells. (**A**) Schematic diagrams with domains of HA genomic-tagged Bni1. The FH domains, the polyPro stretches with the number of consecutive prolines (red) and the position of the first amino acid of each domain/stretch (below) are indicated. (**B**) Western blot for Bni1-HA (anti-HA) and Hxk2 expression in wild-type and *tif51A-1* cells at 25 or 37 °C at the indicated times. (**C**) Translation efficiency of *BNI1-HA* relative to that of *HXK2* in wild-type and *tif51A-1* cells. Protein/mRNA ratios for Bni1 and Hxk2 were calculated by Western and qRT-PCR from same samples. Translation efficiency of *BNI1-HA* was calculated relative to translation efficiency of *HXK2* and represented as a fraction against 25 °C for each strain and from two independent experiments. Data are represented as mean ± SD. Two-tailed student’s t-test analysis: *p < 0.05, **p < 0.01.

Shmoo formation during yeast mating requires Bni1^24^, a formin containing several stretches of consecutive prolines (Fig. 3A). Since we have recently shown that translation of polyPro stretches of Bni1 requires eIF5A^15^, we therefore investigated whether Dm eIF5A also is able to replace Sc eIF5A in the translation of a full-length genomic HA-tagged *BNI1* (Fig. 3A). *BNI1-HA* levels were importantly reduced in *tif51A-1* cells after 4 h at 37 °C^15^, and barely detectable after 6 h (Fig. 3B). However, no reduction in Bni1 protein was observed in *tif51A-1* cells expressing either Sc eIF5A or Dm eIF5A (Fig. 3B). We measured *BNI1-HA* mRNA expression in the same cultures by RT-qPCR and calculated the protein/mRNA ratios as an indication of translation efficiency, which was compared to that of hexokinase 2, a protein with no polyPro motifs. Translation efficiency of *BNI1* was dramatically reduced after the temperature shift to 37 °C in *tif51A-1* cells carrying an empty plasmid whereas wild-type translation efficiencies were observed in Sc eIF5A or Dm eIF5A-transduced *tif51A-1* cells (Fig. 3C). Therefore, fly eIF5A is able to allow the progression of yeast ribosomes through the polyPro encoding codons of *BNI1*, which otherwise stall ribosomes in the absence of endogenous eIF5A^8^. These results indicated that both Sc eIF5A and Dm eIF5A are interchangeable in its regulation of formin translation in yeast.

### **eIF5A regulates the formin Diaphanous during*Drosophila*DC**

**Figure 4:**
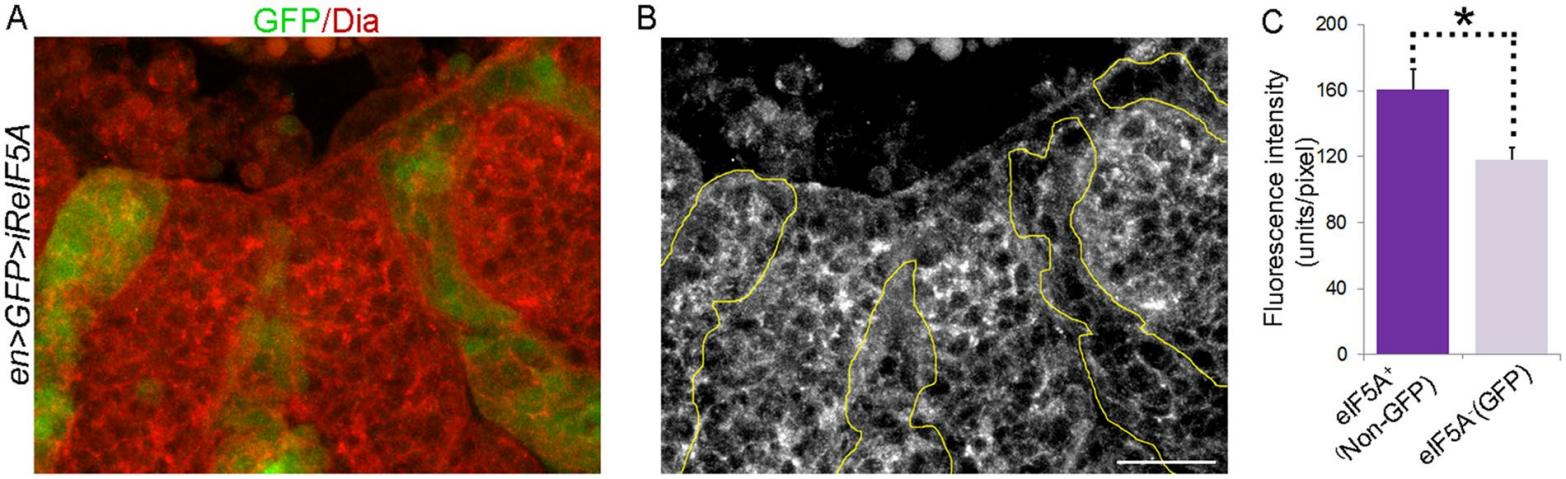
Reduction of Dm *eIF5A* function affects Dia formin levels. (**A,B**) Confocal images of a representative stage 13 *en*-GAL4>UAS-GFP; UAS-*iReIF5A* (*en*>*GFP*>*iReIF5A*) embryo stained with anti-Dia. A lateral view of the epidermis is shown. Anterior it to the left, dorsal is up. (**A**) Overlay of the *en*-GAL4 positive bands, identified by GFP expression, and Dia localization. (**B**) Image of the anti-Dia staining in which yellow lines represent the limits of the *en*-GAL4 positive bands. Decreased Dia levels are observed in cells with reduced *eIF5A* function (GFP marked cells) when compared to nearby non-GFP wild-type cells. Scale bar: 25 µm. (**C**) Quantification of the anti-Dia signal intensity (fluorescence intensity) in *eIF5A* mutant cells (GFP) with respect to control cells (Non-GFP). Data are represented as mean ± SD. Two-tailed student’s t-test analysis: *p < 0.05.

Six formin-encoding genes have been identified in the *Drosophila* genome, including *DAAM* ^25^, *diaphanous* ^26^, *formin3* ^27^, *Fhos* ^28, 29^, *cappuccino* ^30^, and *frl* ^31^, each corresponding to a specific formin protein group (DAAM, DIA, INF, FHOD, FMN and FRL/FMNL, respectively) based on their FH2 domain and additional conserved regions^32^. Among them, only the *diaphanous* (*dia*) gene has been analyzed in DC (reviewed in ref. 32). Embryos lacking *dia* display cell misalignments at the dorsal midline, suggesting a role in coordinating actomyosin contractibility and cell adhesion during DC^33^. Actin cable assembly in *dia* mutant embryos wounded by laser ablation is also inhibited at wound edges^34^, further supporting a role for Dia in actin regulation. Reduced expression of *formin3*, *Fhos*, *cappuccino*, and *frl* in embryonic epidermis using the *arm*-GAL4 and *69B*-GAL4 drivers and the corresponding RNAi lines resulted in fully viable embryos, suggesting that they were not required for DC (not shown). Similar results have been obtained in *DAAM* mutants (J. Mihaly, personal communication). Therefore, we tested whether Dia could be the translational target of Dm eIF5A during DC. Dia protein levels in *arm*-GAL4/UAS-*iReiF5A* or *69B*-GAL4/UAS-*iReiF5A* embryos were apparently lower than in control embryos (*arm*-GAL4/+ and *69B*-GAL4/+) by immunoblot detection (data not shown). The differences were, however, very small, most likely because the strategy used deletes *eIF5A* in only a small fraction of cells. Therefore, we decided to analyze Dia expression at the single cell level in embryos in which *eIF5A* RNAi is driven in a mosaic pattern by the *en*-GAL4 line (Fig. 4A,B). Previous analyses showed that Dia has a dynamic cellular localization during DC, being cortical in elongating epidermal cells^33^. Accordingly we detected Dia by immunofluorescence in the contours of all epidermal cells in both wild-type and *eIF5A* mutant regions. Interestingly, quantifications by image analysis (see Materials and Methods) showed that Dia levels were reduced in *eIF5A* mutant cells compared to nearby wild-type cells (Fig. 4C), consistent with the possibility that Dia translation is regulated by eIF5A.

Since *eIF5A* depletion in the epidermis during DC likely reduce Dia translation, we studied whether an increase of Dia levels in that tissue would be able to suppress the embryonic lethality phenotype of *eIF5A* mutants. First, we generated a fly line harboring two constructs, UAS-*iReIF5A* and UAS-*Dia*, to simultaneously reduce eIF5A endogenous levels and increase Dia levels in a given tissue. This line was crossed with the *69B*-GAL4 epidermal driver and the percentage of embryonic lethality in the progeny was determined. We observed reduced lethality in Dia-overexpressing *eIF5A* mutant embryos (77% dead embryos, n = 816) with respect to that without Dia (94% dead embryos, n = 1,412). Similarly, partial suppression was observed in yeast for the shmooing defects of *eIF5A* mutants when increasing Bni1 formin levels^15^, which could indicate that either higher levels of this formin would be necessary for a complete rescue of the phenotype or that additional proteins involved in DC might also be targets of eIF5A. In summary, phenotypic analysis and immunofluorescence visualization indicate that Dia is a functional target of Dm eIF5A during DC and suggest that defects in actomyosin cable assembly in *eIF5A* mutant embryos are likely the result of low Dia levels.

### Protein levels of Dia and cell migration are regulated by eIF5A in primary mammalian cells

**Figure 5:**
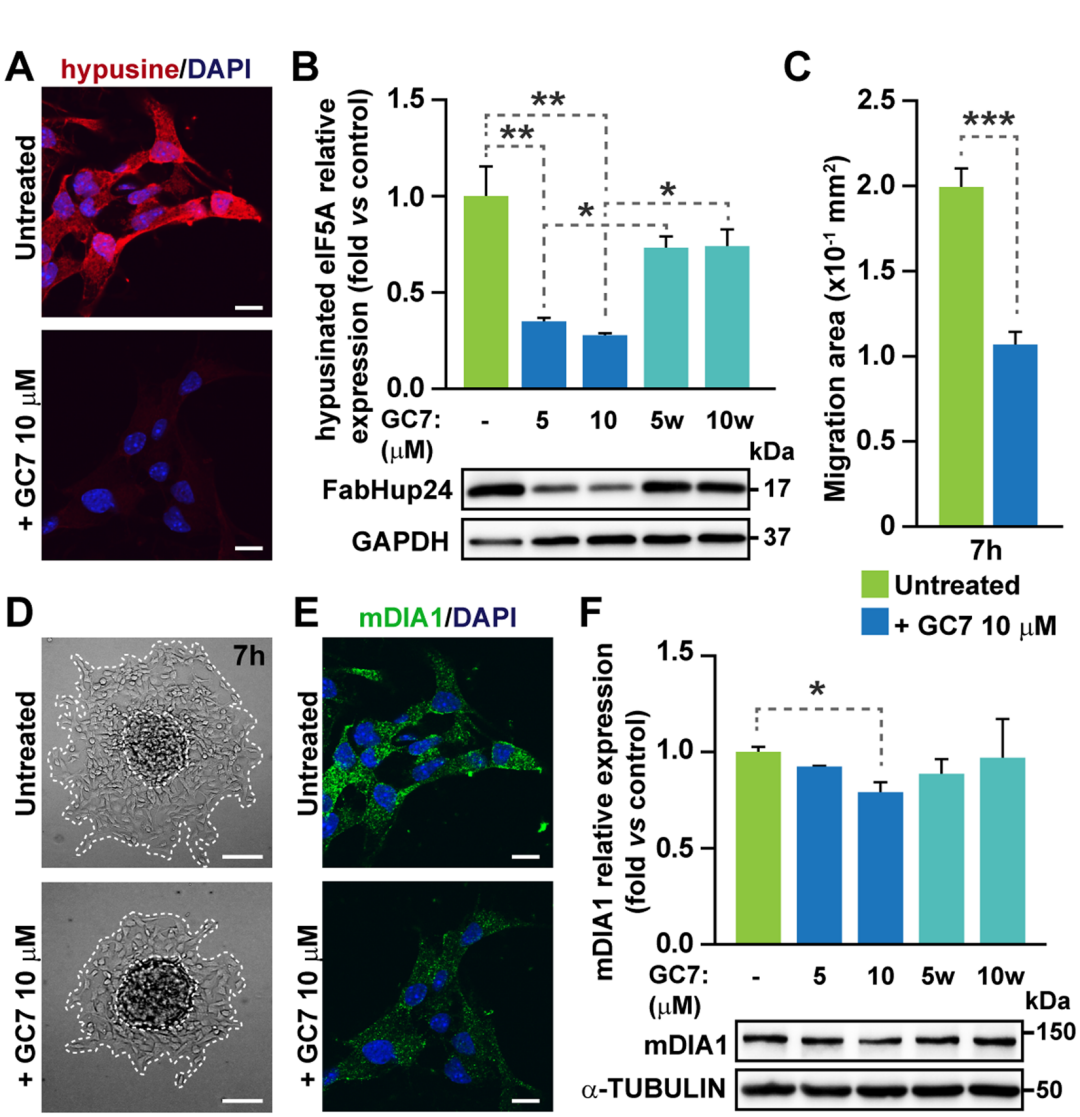
Cell migration and Dia levels are regulated by eIF5A in mouse NSCs. (**A**) Immunocytochemistry for hypusine and DAPI staining in migrating neurospheres with or without 10 μM GC7. (**B**) Western blot for hypusinated eIF5A (FabHpu24) and control GAPDH in NSCs treated during 3 days with GC7 (5 and 10 μM) and then washed and cultured for 3 more days (5w and 10w) (n = 3). (**C**) Quantification of the migration area of individual neurospheres 7 h after treatment (32 to 44 neurospheres analyzed). (**D**) Representative images of the neurosphere migration assay. Migration front and original neurosphere are marked with dashed lines. (**E**) Immunocytochemistry for mDIA1 and DAPI staining in untreated and GC7 treated neurospheres in migration conditions. (**F**) Western blot for mDIA1 and control α-TUBULIN in NSCs treated for 3 days and then washed (n = 3). Two-tailed Student’s t-test analysis: *p < 0.05, **p < 0.01, ***p < 0.001. Scale bars: **A** and **E**, 10 μM; **D**, 100 μm.

Mammalian Dia1 (mDia1) catalyzes actin polymerization and regulates microtubule dynamics, playing an important role in cell polarization, adhesion and migration downstream of GTPase RhoA^35, 36^. A requirement for eIF5A in cytoskeletal-mediated processes such as cell migration has also been reported^37^, however a link between mDia and eIF5A has not been established. We found that NSCs isolated from the subependymal zone of the adult mouse brain and grown as neurospheres express detectable levels of eIF5A1 but not eIF5A2 (mRNA expression normalized to *Gapdh*, in mean arbitrary units ± SD × 10^−3^: *Eif5a1*, 132 ± 4*; Eif5a2*, 0.02 ± 0.09; n = 3); in addition, we also found detectable levels of mDia mRNA (45 ± 3; n = 3). Therefore, we decided to test whether mDia protein could be regulated by eIF5A in NSCs. Hypusination is a post-translational modification selectively found in eIF5A that is essential for its function^6^. A 3-day treatment of neurospheres with the cell-permeable inhibitor of deoxyhypusine synthase GC7 at 5 or 10 µM did not affect cell viability (not shown), but resulted in a 3-fold reduction in the levels of hypusinated eIF5A that could be completely reversed by washing the drug out for 3 additional days (Fig. 5A,B). Individual neurospheres of similar sizes that had been grown with or without 10 µM GC7 for 5 days were then plated onto Matrigel. Quantitation of cell dispersion from each neurosphere at different times after plating indicated that inhibition of hypusination reduces the extent of migration (Fig. 5C,D). More interestingly, mDia1 levels were decreased by inhibition of eIF5A hypusination and rescued after drug wash-out (Fig. 5E,F) without changes in mRNA expression (mean fold-change relative to control condition ± SD: 1.00 ± 0.04 after 10 µM GC7 and 1.03 ± 0.09 after wash-out, n = 3).

Our results together indicate that eIF5A is required for Dia translation in both vertebrate and invertebrate cells. We demonstrate that structural and sequence conservation of eIF5A across evolution correlates with its conserved role in cytoskeletal dynamics through the regulation of formin translation. Actin nucleation is a tightly regulated process^1^, therefore the unique modulation of eIF5A activity by polyamine-dependent hypusination opens exciting possibilities for the pharmacological targeting of cytoskeletal remodeling in different biological systems.

## Materials and Methods

### *Drosophila* strains

Fly stocks *69B*-GAL4 and *arm*-GAL4 driver lines, UAS-*iReIF5A*, UAS-*iRDAAM*, UAS-*iRcapu*, UAS-*iRform3*, UAS-*iRfhos*, and UAS-*iRfrl* RNAi lines, and UAS-*Dia*-GFP line were obtained from the Bloomington *Drosophila* Stock Center (BDSC). The stock harboring both UAS-*Dia*-GFP and UAS-*iReIF5A* constructs was generated by standard genetic techniques. Flies were grown on standard media at 25 °C. All crosses were performed at 25 °C except those using UAS-*iRcapu*, UAS-*iRform3*, UAS-*iRfhos*, and UAS*-iRfrl* RNAi lines which were performed at 29 °C.

### Cuticle preparations and lethality assays in *Drosophila* embryos

For cuticle analyses, embryos were collected and processed as reported^19^ and examined using Nomarski DIC optics. Lethality assays were performed at 25 and 29 °C, except when using UAS-*iReIF5A* and UAS-*iReIF5A*; UAS-*Dia*-GFP that were performed only at 25 °C.

### *Drosophila* embryos and imaginal disc immunofluorescence

For immunohistochemistry, embryos were fixed with 3.7% formaldehyde diluted in phosphate buffer saline (PBS) and 1:1 heptane-methanol for devitellinization. Third instar larval wing imaginal discs were dissected, fixed and stained as described^38^. Primary antibodies used were: rabbit anti-Dia (1:500^39^, mouse anti-GFP (1:250, Roche) and rabbit anti-eIF5A (1:100, Abcam). Secondary antibodies coupled to fluorochromes were purchased from Invitrogen and used at a 1:200 dilution. For phalloidin staining, embryos were fixed with 4% paraformaldehyde in PBS and devitellinization was done with in 1:1 heptane-80% ethanol. After washing in 100% ethanol, embryos were incubated twice in PBS with Triton-X100 (PBS-Tx)−1% BSA for 10 min each time, twice in PBS-Tx-2% BSA for 10 min each time and 1 h in *Alexa Fluor 568 phalloidin* reagent (1:20, Invitrogen). Phalloidin incubation was followed by 15 min incubations of PBS-Tx with decreasing concentrations of BSA (2-0%). Specimens were mounted in Mowiol and images were acquired either on a LeicaTCS-NT confocal laser-scanning microscope or on an Olympus FV1000MPE confocal microscope. Several Z sections of each embryo were taken and processed with ImageJ. Imaginal discs images were acquired in a Leica DMI2500 optical fluorescence microscope. For quantification of fluorescence intensity in *en*>GFP> *iReIF5A* embryos, images of anti-Dia stainings were converted to grayscale. The Mean Gray Values of ten regions of interest of similar areas were measured per segment with ImageJ. The fluorescence intensity was calculated as the average of the Mean Gray Values obtained from three *eIF5A* mutant and three control segments.

### Yeast strains, growth conditions and plasmids

*Saccharomyces cerevisiae* haploid strains wild-type BY4741 (MATa his3∆0 leu2∆0 met15∆0 ura3∆0) and temperature-sensitive eIF5A mutants *tif51A-1* and *tif51A-3* derivatives of the BY4741 strain^23^ were grown in liquid YPD or SC complete medium when carrying plasmids at 25 °C until the exponential phase and then treated as indicated. The Sc and Dm *eIF5A* genes were cloned in the yeast centromeric heterologous expression plasmid p416TEF (URA3 marker), under the control of the constitutive TEF promoter and using the *CYC1* terminator (PA300 and PA304 plasmids, respectively). For this, we used a GAP repair strategy^40^. The ORF of the yeast *TIF51A* gene (encoding the essential copy of eIF5A) was PCR amplified using primers GAPR 5′-eIF5A-Sc (5′-GTTTTCTAGAACTAGTGGATCCCCCGGGCTGCAGGAATTCGATATGTCTGACGAAGAACA-3′) and GAPR 3′-eIF5A-Sc (5′-ACTAATTACATGACTCGAGGTCGACGGTATCGATAAGCTTGATTTAATCGGTTCTAGCAG-3′) and yeast genomic DNA as template. The Dm *eIF5A* unique gene was amplified with primers GAPR 5′-eIF5A-Dm (5′-GTTTTCTAGAACTAGTGGATCCCCCGGGCTGCAGGAATTCGATATGGCTGAGTTGGACGA-3′) and GAPR 3′-eIF5A-Dm (5′-ACTAATTACATGACTCGAGGTCGACGGTATCGATAAGCTTGATCTATTTGTCCAGAGCAG-3′) using the GH16179 cDNA clone from the Drosophila Genomics Resource Center (DGRC) EST collection as template. PCR cassettes were co-transformed with *Eco*RI linearized p416TEF plasmid^41^ in BY4741 yeast cells and cells with GAP repaired plasmids were selected in SC-ura media. Plasmids were recovered from yeast cells and directly transformed in *E. coli* competent cells, then plasmids were extracted from bacteria and the correct cloning was confirmed by DNA sequencing.

### Shmooing analysis in yeast cells

Strains were grown in experimental temperatures and treated with 10 μg/ml of α-factor (Sigma) for 2 h. After incubation for 4 h at the indicated temperatures, cells were visualized by microscopy and images were taken (Axioskop 2 Florescence Microscope and AxioCam MRm, Zeiss Inc.). Approximately 200 cells were analyzed *per* sample from at least two independent experiments.

### NSCs culture and migration assays

Subependymal NSCs were isolated from 2 month-old C57BL/6J mice and cultured as described^42^. Viability was analyzed using a commercial MTS assay (Promega). Deoxyhypusine synthase inhibitor GC7 (Calbiochem) was dissolved in sterile distilled water. For migration assays, neurospheres were individually selected according to their size and plated on Matrigel®-coated coverslips. Migration area was measured with ImageJ software and normalized to the initial neurosphere area.

### NSCs immunofluorescence

Neurospheres were fixed with 2% paraformaldehyde in 0.1M phosphate buffer, incubated with mouse anti-mDIA1 (1:50, BD Bioscience) or rabbit anti-hypusine (1:200, Millipore) overnight and then with Alexa Fluor® 488 donkey anti-mouse (1:800, Molecular Probes) or Cy3 donkey anti-rabbit (1:800, Jackson Immunoresearch) secondary antibodies for 1 h. DAPI (1 μg/ml) was used to counterstain nuclei. Pictures were taken using a confocal microscope (Olympus FV10) and all images were processed equally with Photoshop.

### Western blot

Western blot analyses with yeast extracts were performed with anti-HA 12C5A (1:1000, Roche) and anti-Hxk2 (1:20000^43^) as described^44^. Denatured protein extracts from neurospheres were separated by 15% (for hypusine detection) and 8% (for mDia1) SDS-PAGE and transferred to a nitrocellulose membrane. Antibodies used were: mouse anti-mDIA1 (1:100; BD Bioscience), mouse anti-GAPDH (1:5000, Millipore), mouse anti-α-tubulin (1:5000, Sigma), rabbit anti-hypusine, FabHpu24 (1:600, Genentech), HRP goat anti-mouse (1:5000, Dako) and HRP goat anti-rabbit (1:5000, Santa Cruz). Band intensities of immunoblots were detected and quantified with the ImageQuant LAS 4000 mini image analyzer (GE Healthcare).

### Quantitative RT-PCR

RNA extraction, reverse transcription and quantitative PCR (qRT-PCR) analyses in yeast were used to quantify *BNI1-HA* and *HXK2* mRNA amount and normalized against endogenous *ACT1* expression as described^45^. Translation efficiencies of *BNI1-HA* and *HXK2* were estimated as the protein/mRNA ratio, using the Western and RT-qPCR data (normalized against *ACT1* expression) obtained from cell samples of the same experiment. *BNI1-HA* translation efficiency is expressed relative to *HXK2* translation efficiency and represented as a fraction against 25 °C for each strain. For NSCs, RNA was isolated using RNeasy Mini Kit (Qiagen), cDNA was synthesized using PrimeScript™ RT reagent Kit (Takara) and qRT-PCR was performed using TaqMan® probes (Life Technologies). For qRT-PCR in yeast, BNI1-3/ BNI1-4, HXK2-1/HXK2-2, and ACT1-1/ACT1-2 primers were used, which are described in ref. 15. For qRT-PCR in NSCs, the following probes were used: Eif5a1 (Mm01971736_g1), Eif5a2 (Mm00812570_g1), Cyb5r3 (Mm00504077_m1) and Gapdh (Mm99999915_g1).
